# Antioxidant and Neuroprotective Effects of Fucoxanthin and Its Metabolite Fucoxanthinol: A Comparative In Vitro Study

**DOI:** 10.3390/cimb46060357

**Published:** 2024-06-14

**Authors:** Letizia Pruccoli, Martina Balducci, Barbara Pagliarani, Andrea Tarozzi

**Affiliations:** 1 Department for Life Quality Studies, University of Bologna, 47921 Rimini, Italy; letizia.pruccoli2@unibo.it (L.P.); martina.balducci8@unibo.it (M.B.); barbara.pagliarani2@unibo.it (B.P.); 2 Biostructures and Biosystems National Institute (INBB), 00136 Rome, Italy

**Keywords:** fucoxanthin, fucoxanthinol, carotenoids, antioxidants, oxidative stress, neuroprotection, Nrf2, amyloid beta, 6-hydroxydopamine

## Abstract

Fucoxanthin is the most abundant carotenoid found in marine brown algae that exhibits several healthy properties. Dietary fucoxanthin is metabolized in the intestine, plasma, and other tissues to various metabolites, including fucoxanthinol. In this regard, the contribution of fucoxanthinol to the healthy properties of its precursor, fucoxanthin, against pathogenetic events associated with neurodegenerative diseases remains unexplored. Here, we evaluated and compared the antioxidant and neuroprotective effects of the carotenoids fucoxanthin and fucoxanthinol in in vitro models of Alzheimer’s (AD) and Parkinson’s (PD) disease. Neuronal SH-SY5Y cells were used to evaluate the antioxidant properties of the carotenoids against ABTS radical in the membrane and cytoplasm and oxidative stress elicited by *tert*-butyl hydroperoxide using the 2′,7′-dichlorodihydrofluorescein diacetate probe. We also assessed the ability of the carotenoids to increase the glutathione (GSH) and activate the Nrf2/Keap1/ARE pathway using the monochlorobimane probe and western blotting method, respectively. The neuroprotective effects of the carotenoids against the neurotoxicity generated by oligomers of Beta-Amyloid (1–42) peptide (OAβ) and 6-hydroxydopamine (6-OHDA), which are neurotoxins of AD and PD, respectively, were finally evaluated in the same neuronal cells using the thiazolyl blue tetrazolium bromide assay. Both carotenoids could reach the cytoplasm, which explains the mainly free radical scavenging activity at this level. Notably, fucoxanthinol had higher and lower antioxidant activity than fucoxanthin at extracellular and cellular levels. Although studied carotenoids exerted the ability to activate the Nrf2/Keap1/ARE pathway, leading to an increase of intracellular GSH, our results suggested that the antioxidant activity of the carotenoids could be mainly attributed to their radical scavenging activity in neuronal membrane and cytoplasm, where they accumulate. Fucoxanthinol also shared similar neuroprotective effects as fucoxanthin against the neurotoxicity generated by OAβ and 6-OHDA, suggesting a potential neuroprotective contribution to the action of fucoxanthin administered as a food supplement in in vivo experimental models. These results encourage further research to evaluate the bioavailability of fucoxanthinol and other metabolites of fucoxanthin at the brain level to elucidate the dietary neuroprotective potential of fucoxanthin.

## 1. Introduction

**Figure 1:**
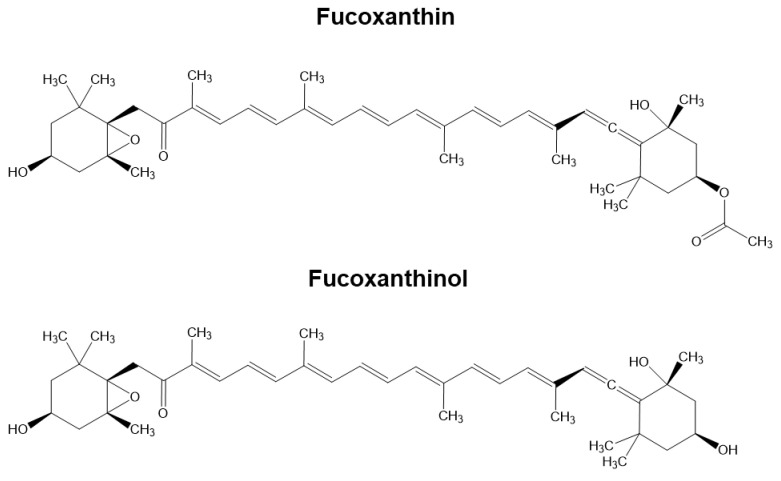
Chemical structure of carotenoids.

Fucoxanthin is the most abundant carotenoid found in edible marine brown algae. Thanks to its unique chemical structure, fucoxanthin exhibits numerous healthy properties in diabetes, obesity, cardiovascular diseases, cancer, and neurodegenerative diseases [1]. In this regard, recent studies have shown the ability of fucoxanthin to counteract amyloid protein aggregation, oxidative stress (OS), neuroinflammation, neuronal death, and neurotransmission dysregulation in different experimental models of psychiatric disorders [2], acute brain injury [3,4], and Alzheimer’s (AD) [5,6,7,8,9,10] and Parkinson’s (PD) disease [11,12,13]. Dietary fucoxanthin is readily hydrolyzed to fucoxanthinol by digestive enzymes of lipase and cholesterol esterase and further converted to amarouciaxanthin A by NAD(P)+-dependent dehydrogenases in the gastrointestinal tract and liver, respectively [14]. Bioavailability studies demonstrated the distribution and accumulation of the metabolite fucoxanthinol in various tissues, including the liver, lung, kidney, heart, spleen, epididymal, and adipose tissue, after the oral administration of dietary fucoxanthin in mice [15,16]. The bioavailability of fucoxanthin was also studied in human volunteers after the oral administration of kombu algae containing fucoxanthin. The study found fucoxanthinol but not fucoxanthin in human plasma. This suggests that fucoxanthin is metabolized in the gastrointestinal tract and absorbed into the blood as its deacetylated metabolite, fucoxanthinol [17,18]. A further bioavailability study in rats identified plasmatic levels of fucoxanthinol higher than fucoxanthin after either intravenous or intragastric administrations, indicating that the low bioavailability of fucoxanthin could be ascribed its fast metabolism in the intestine, plasma, and other tissues to fucoxanthinol [19]. Fucoxanthin has been shown to prevent various neurodegenerative processes in animal models, indicating that it may have healthy effects on the brain. In the mouse model of traumatic brain injury, fucoxanthin produced neuroprotective effects through the nuclear factor erythroid 2-related factor 2 (Nrf2)/antioxidant response element (ARE) pathway when administered via intra-cerebroventricular injection or intragastric administration. The authors suggested that the oral intake of fucoxanthin could lead to the effective concentration of fucoxanthinol in the brain, preventing neuronal injury [4]. Remarkably, the intragastric administration of fucoxanthin in mice prevented the inflammation in the hippocampus and frontal cortex induced by lipopolysaccharide via the adenosine monophosphate-activated protein kinase-nuclear factor-κB pathway, as well as attenuated cognitive impairments evoked by Beta-Amyloid (Aβ) oligomers or scopolamine [2,5,10]. Since fucoxanthin has not been detected in tissue, its healthy effects may be expressed by fucoxanthin-derived metabolites, including fucoxanthinol, as active compounds in the body [16]. Several in vitro studies show that fucoxanthinol shares some antioxidant and anti-inflammatory mechanisms in animal models fed dietary fucoxanthin [20,21,22,23,24]. However, the antioxidant and neuroprotective effects of the metabolite fucoxanthinol against OS and neuronal death in AD and PD remain unexplored. Here, we evaluated the ability of the metabolite fucoxanthinol to counteract OS, as well as the cell death induced by oligomers of Aβ_1–42_ peptides, soluble aggregates of Aβ peptide involved in the pathogenesis of AD, or 6-hydroxydopamine hydrochloride (6-OHDA), a neurotoxin of PD, in human neuronal SH-SY5Y cells. In particular, we compared fucoxanthinol and its metabolic precursor, fucoxanthin, which has a similar chemical structure (Figure 1). This comparison aimed to examine the relationship between the chemical structure of these carotenoids and their ability to provide antioxidant and neuroprotective activity at the neuronal level.

## 2. Materials and Methods

### 2.1. Chemicals

2,2′-Azino-bis (3-ethylbenzothiazoline-6-sulfonic acid) diammonium salt (ABTS), 2′,7′-dichlorodihydrofluorescein diacetate (DCFH-DA), 3-(4,5-dimethyl-2-thiazolyl)-2,5-diphenyl-2H-tetrazolium bromide (MTT), fucoxanthin, fucoxanthinol, 6-OHDA, monochlorobimane (MCB), and *tert*-butyl hydroperoxide solution (*t*-BuOOH) were obtained from Sigma-Aldrich (Sigma-Aldrich, St. Louis, MO, USA). PureLink RNA Mini Kit, SuperScript VILO Master Mix and SYBR Select Master Mix were obtained from Life Technologies Italia. Aβ_1–42_ peptide was obtained from AnaSpec (AnaSpec, Fremont, CA, USA). All chemicals used in the experiments were of analytical grade.

### 2.2. Cell Culture and Preparation of Carotenoid Solutions

The human neuroblastoma SH-SY5Y cell line (catalog no. 94030304) was supplied by the European Collection of Authenticated Cell Cultures (ECACC, UK Health Security Agency, Porton Down, Salisbury, UK). Cells were certified free of mycoplasma and authenticated using the supplier’s short tandem repeat method. Cells were grown in Dulbecco’s modified Eagle Medium supplemented with fetal bovine serum (10%), L-glutamine (2 mM), penicillin (50 U/mL), and streptomycin (50 μg/mL) in a humidified incubator at 37 °C with 5% CO_2_. To perform experiments with SH-SY5Y cells, we prepared stock solutions of fucoxanthin and fucoxanthinol in dimethyl sulfoxide (DMSO) at 20 mM. We diluted the stock solutions in a complete medium to achieve the required carotenoid concentrations, ensuring that DMSO was below 0.1%.

### 2.3. Determination of Neuronal Viability

The viability of the neuronal cells was assessed using the MTT assay, as previously described [25]. SH-SY5Y cells were placed in a 96-well plate, with 2 × 10^4^ cells per well, and incubated for 24 h. After that, the cells were treated with various concentrations of carotenoids (2.5–40 µM) for another 24 h at 37 °C in 5% CO_2_. The treatment medium was subsequently replaced with MTT in Hank’s Balanced Salt Solution (HBSS) at a concentration of 0.5 mg/mL and then incubated for 2 h at 37 °C in 5% CO_2_. After washing the cells with HBSS, formazan crystals were solubilized in isopropanol. Finally, the amount of formazan was measured at 570 nm (reference filter 690 nm) using the multitask plate reader VICTOR™ X3 (PerkinElmer, Waltham, MA, USA).

### 2.4. Determination of Total Antioxidant Activity (TAA)

The TAA of the carotenoids was assessed using ABTS assay, as previously described [25]. A 2 mM ABTS solution was mixed with potassium persulfate (7 mM) and kept in the dark at room temperature for 24 h to generate ABTS radicals. The ABTS solution was then diluted with phosphate-buffered saline (PBS) before being used to achieve an absorbance value of 0.70 at 734 nm. Subsequently, 1 mL of the diluted ABTS solution was added to 10 µL of the studied carotenoids at a concentration of 5 µM; the absorbance at 734 nm was measured after 1 min. TAA was expressed as the equivalent of micromoles of Trolox per milliliter of carotenoid solution.

### 2.5. Determination of TAA in Membrane and Cytoplasm of SH-SY5Y Cells

The TAA of the membrane and cytoplasm of SH-SY5Y cells treated with carotenoids was evaluated using ABTS assay as previously described [25]. The cells were placed in 60 mm dishes at 2 × 10^6^ cells per dish. After incubating for 24 h, the cells were treated with 5 µM fucoxanthin and fucoxanthinol for 2 h at 37 °C in 5% CO_2_. After incubation, the cells were washed three times with cold PBS and softly detached from the dish using a cell lifter. After collecting the cells in 1 mL of PBS, they were centrifuged at 10,000 rpm for 10 min at 4 °C. The supernatant was removed, and the cells were again washed with 1 mL of PBS. This washing process was repeated twice, after which the pellet was reconstituted in 600 µL of lysis buffer containing Triton X-100 0.05%. After homogenizing, the cells were left to stand at 4 °C for 30 min. The mixture was centrifuged at 14,000 rpm for 15 min at 4 °C to extract the cytoplasmic fraction. The pellet was then dissolved in 400 µL of lysis buffer containing Triton X-100 1% to obtain the membrane fraction. Membrane and cytoplasmic cell fractions were kept red at −20 °C. Small amounts were taken out to determine the protein concentration using the Bradford method. The ABTS assay measured the antioxidant activity of carotenoids on membrane and cytoplasmic fractions. ABTS decolorization was compared to a standard curve of Trolox, a water-soluble analog of vitamin E, to calculate the TAA of membrane and cytosolic fractions. The Trolox standard curve was prepared using concentrations ranging from 50 to 150 µM. The TAA was expressed as the equivalent of micromoles of Trolox per milligram of protein.

### 2.6. Determination of Antioxidant Activity in SH-SY5Y Cells

The antioxidant activity of carotenoids was evaluated in SH-SY5Y cells using the DCFH-DA assay, as previously described [25]. DCFH-DA is a fluorogenic probe for the detection of intracellular ROS. The cell-permeable DCFH-DA diffuses into cells and is deacetylated by cellular esterases to form 2′,7′-dichlorodihydrofluorescein. Then, in the presence of ROS, it is oxidized to 2′,7′-dichlorofluorescein, which is highly fluorescent.

SH-SY5Y cells were placed in a 96-well plate at 3 × 10^4^ per well and incubated for 24 h. After incubation, the medium was removed, and 100 µL of the fluorescent probe DCFH-DA in PBS (10 g/mL) was added to each well. The plate was then incubated for 30 min at room temperature. After incubation, the DCFH-DA solution was replaced with 100 µL of *t*-BuOOH (100 µM) and 100 µL of the studied carotenoids (2.5–5 µM). The plate was then incubated for another 30 min, after which the reactive oxygen species (ROS) formation was measured using the multitask plate reader VICTOR™ X3 (excitation at 485 nm and emission at 535 nm). Another set of SH-SY5Y cells was placed in a 96-well plate at 2 × 10^4^ cells per well, incubated for 24 h, and treated with carotenoids (2.5–5 µM) for 24 h. After incubation, the treatment medium was discarded, and 100 µL of DCFH-DA in PBS (10 g/mL) was added to each well. The plate was then incubated for 30 min at room temperature; the DCFH-DA solution was replaced with 100 µL of *t*-BuOOH (100 µM). The ROS formation was measured after 30 min of incubation, as described above. Data are expressed as a fold increase in ROS formation induced by *t*-BuOOH.

### 2.7. Determination of Glutathione Levels

The glutathione (GSH) level was evaluated in SH-SY5Y cells using the MCB assay, as previously described [26]. MCB is a fluorescent probe that reacts with several low molecular weight intracellular thiols, including glutathione. The cells were placed in a black 96-well plate at 2 × 10^4^ cells per well. After 24 h of incubation, the cells were treated with fucoxanthin and fucoxanthinol at a concentration of 5 µM for 24 h at 37 °C in 5% CO_2_. After incubation, the treatment medium was removed, and 100 µL of the fluorescent probe MCB in PBS (50 µM) was added to each well. After 30 min of incubation at 37 °C, GSH levels were measured (excitation at 355 nm and emission at 460 nm) using the multitask plate reader VICTOR™ X3 (PerkinElmer). Data were expressed as the micromolar concentration of GSH obtained using a GSH standard curve.

### 2.8. Determination of Keap1 and Nrf2 Protein Levels

Keap1 and Nrf2 protein levels were assessed in SH-SY5Y cells using western blotting, as previously described [27]. The cells were placed in 60 mm dishes at 2 × 10^6^ cells per dish, incubated for 24 h, and then treated with carotenoids (2.5–5 µM) for 3 h at 37 °C in 5% CO_2_. After incubation, the cells were pelleted, and a complete lysis buffer containing leupeptin (2 µg/mL), phenylmethanesulfonyl fluoride (100 µg/mL), and a cocktail of protease/phosphatase inhibitors (100×) was added. A small amount of the lysate was used to determine the protein concentration using the Bradford assay. The protein lysates (50 μg per sample) were separated by 12% SDS polyacrylamide gels (Bio-Rad Laboratories, Hercules, CA, USA) and transferred onto 0.45 μm nitrocellulose membranes. The membranes were then probed with primary Keap1 (1:1000; catalog no. 8047, Cell Signaling Technology, Danvers, MA, USA) or Nrf2 antibody (1:1000; catalog no. sc-722, Santa Cruz Biotechnology, Dallas, TX, USA) and secondary antibody. Enhanced chemiluminescence reagents (Pierce, Rockford, IL, USA) were used to detect targeted bands. The same membranes were probed with an anti-β-Actin antibody (1:1000; catalog no. A1978, Sigma-Aldrich) and a secondary antibody. Densitometry analysis was performed using the Image Lab software (version 5.2, Bio-Rad Laboratories). The results were expressed as the ratio between Keap1 or Nrf2 and β-Actin protein levels.

### 2.9. Determination of Nrf2 and Nrf2-Target Gene Expression

Nrf2 and Nrf2-target gene expression was evaluated using quantitative real-time polymerase chain reaction (qRT–PCR), as previously described [28]. SH-SY5Y cells were placed in 60 mm dishes at 2 × 10^6^ cells per dish and incubated for 24 h at 37 °C in 5% CO_2_. After that, the cells were treated with carotenoids at a concentration of 5 µM for another 24 h. At the end of incubation, the cells were centrifugated at 6000 rpm for 5 min at 4 °C to obtain a pellet, and total RNA was isolated using a PureLink RNA Mini Kit (Life Technologies, Carlsbad, CA, USA). First-strand cDNA synthesis was performed with 1 µg of total RNA using the SuperScript VILO MasterMix (Invitrogen, Carlsbad, CA, USA). PCR reactions of each sample were performed in triplicate in a final volume of 20 µL in a 96-well plate. The PCR mixture was prepared by combining 2 µL of cDNA (10 ng/µL), 10 µL of SYBR Select Master Mix (Invitrogen), and primers at a final concentration of 300 nM. The mixture was then amplified with the following conditions: 3 min at 95 °C, followed by 40 amplification cycles of 3 s at 95 °C and 30 s at 60 °C. After this reaction, a melting curve analysis was performed (65–95 °C) with five acquisitions/°C. TBP and B2M were used as reference genes, and the experimental results were calculated using the 2^−(ΔΔCq)^ method. The primers used were as follows: sense, 5′-CATCTACAAACGGGAATGTCTG-3′ and antisense, 5′-AGTGGATCTGCCAACTACTC-3′ for nuclear factor erythroid 2-related factor 2 (Nrf2); sense, 5′-ATTTGAATTCGGGCGTCTGC-3′ and antisense, 5′-GGGATCCACGGGGACATGA-3′ for NAD(P)H quinone dehydrogenase 1 (NQO1); sense, 5′-TCTCCCTCATCTACACCAACTATG-3′ and antisense, 5′-AGGTCTTGCCTCCCTGGT-3′ for glutathione S-transferase P1 (GSTP1); sense, 5′-CACATCACAGCTCCCCACCA-3′ and antisense, 5′-TGCACAGGAGCCAAGAGTGAA-3′ for TATA-Box Binding Protein (TBP); and sense, 5′-CTTTCCATTCTCTGCTGGATGACG-3′ and antisense, 5′-GCGGGCATTCCTGAAGCTGACAGCA-3′ for Beta 2 microglobulin (B2M).

### 2.10. Determination of Neuroprotective Activity against Aβ_1–42_ Oligomers

The Aβ_1–42_ peptide aggregation to oligomeric form (AβO) was prepared as previously described [29]. The neuroprotective activity of carotenoids against AβO-induced neurotoxicity was evaluated in SH-SY5Y cells using the MTT assay. SH-SY5Y cells were placed in a 96-well plate at 3 × 10^4^ cells per well, incubated for 24 h, and then treated with carotenoids (5 µM) in the presence of AβO (10 µM) for 4 h at 37 °C in 5% CO_2_. Next, the treatment medium was replaced with MTT in HBSS (0.5 mg/mL) for 2 h at 37 °C in 5% CO_2_. The formazan crystals were solubilized in isopropanol after washing with HBSS. The amount of formazan was then determined at 570 nm (reference filter 690 nm) using the multitask plate reader VICTOR™ X3 (PerkinElmer). Data were expressed as percentages relative to untreated cells.

### 2.11. Determination of Neuroprotective Activity against 6-OHDA

The neuroprotective activity of carotenoids against 6-OHDA-induced neurotoxicity was evaluated in SH-SY5Y cells using the MTT assay. SH-SY5Y cells were placed in a 96-well plate at 3 × 10^4^ cells per well and incubated for 24 h. They were then treated with carotenoids (5 µM) for 24 h at 37 °C in 5% CO_2_. After that, the cells were treated with 6-OHDA (100 μM) for 2 h, and the treatment medium was replaced with MTT in HBSS (0.5 mg/mL) for 2 h at 37 °C in 5% CO_2_. The formazan crystals were solubilized in isopropanol after washing with HBSS. The amount of formazan was then determined at 570 nm (reference filter 690 nm) using the multitask plate reader VICTOR™ X3 (PerkinElmer). Data were expressed as percentages relative to untreated cells.

### 2.12. Statistical Analysis

Data were reported as mean ± standard deviation (SD) of at least three independent experiments. Statistical analysis was realized using PRISM 5 software (GraphPad Software, La Jolla, CA, USA). Student’s *t*-test and one-way ANOVA with Tukey post hoc test were used to determine statistical significance (*p* < 0.05).

## 3. Results and Discussion

### 3.1. Antioxidant Activity of Carotenoids

**Figure 2:**
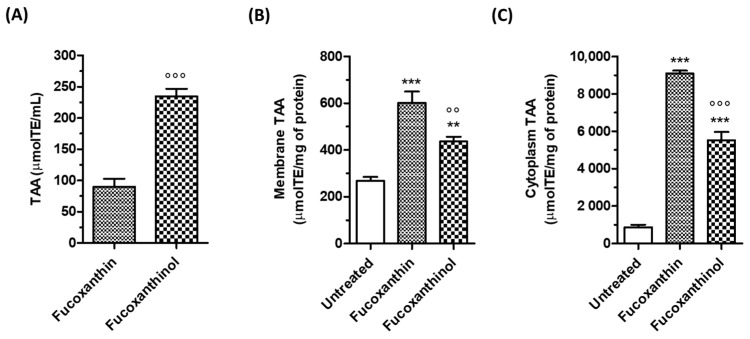
TAA of carotenoids. (**A**) Data are expressed as equivalent of micromoles of Trolox and reported as the mean ± SD of three independent experiments (°°° *p* < 0.001 vs. fucoxanthin, Student’s *t*-test); (**B**,**C**) SH-SY5Y cells were treated for 2 h with the studied carotenoids at a concentration of 5 µM. At the end of treatment, the ABTS radical scavenging activity was evaluated in the membrane and cytoplasmic fractions using the ABTS assay. Data are expressed as equivalent of the micromoles of Trolox per milligram of protein and shown as mean ± SD of three independent experiments (°° *p* < 0.05 and *°°° p <* 0.001 vs. cells treated with fucoxanthin; ** *p* < 0.01 and *** *p* < 0.001 vs. untreated cells, one-way ANOVA with Tukey post hoc test).

The TAA of the carotenoids fucoxanthin and fucoxanthinol, in terms of ability to scavenge free radicals, was evaluated using ABTS in cell-free assay and expressed as equivalent to micromoles of Trolox. The ABTS radical scavenging activity of fucoxanthinol was significantly higher than that of the fucoxanthin at the same concentration of 5 µM (Figure 2A). It is known that the two hydroxyl groups in the ring structure of fucoxanthin can donate electrons or hydrogen atoms, which leads to the free radical scavenging and antioxidant activities of the carotenoid [30,31]. Therefore, the higher ABTS scavenging activity exhibited by fucoxanthinol may be ascribed to three hydroxyl groups compared with two groups in fucoxanthin [20]. To assess the effectiveness of fucoxanthinol and its precursor fucoxanthin as antioxidants in SH-SY5Y cells, we first determined a range of carotenoid concentrations that did not cause neurotoxicity. The treatment of SH-SY5Y cells with concentrations of up to 10 µM did not affect neuronal viability using the MTT assay (Supplementary Figure S1). A concentration range of 2.5–5 µM was chosen for all the following experiments. To evaluate the ability of the studied carotenoids to exert their antioxidant activity at the neuronal level, we measured the TAA, expressed as equivalent of micromoles of Trolox for milligram of protein, of membrane and cytoplasmic-enriched fractions of SH-SY5Y cells treated with carotenoids (5 μM) for 2 h. At the end of incubation, we separated membrane and cytoplasmic cell fractions and submitted them to the ABTS assay. As shown in Figure 2B,C, both the carotenoids significantly enhanced the TAA of SH-SY5Y cell membrane and cytoplasm. By comparing the TAA of the carotenoids, fucoxanthin showed a higher TAA in both membrane and cytoplasm than fucoxanthinol. In this regard, it is plausible that the presence of an acetyl group in the terminal ring of fucoxanthin instead of the hydroxyl group in fucoxanthinol contributes to increasing the lipophilicity of fucoxanthin and its ability to cross the cell membrane and reach the cytoplasm explaining the higher ABTS scavenging activity at these cellular comparts. Taken together, these results show that the metabolite fucoxanthinol has a higher and lower antioxidant activity than fucoxanthin at extracellular and cellular levels, respectively.

**Figure 3:**
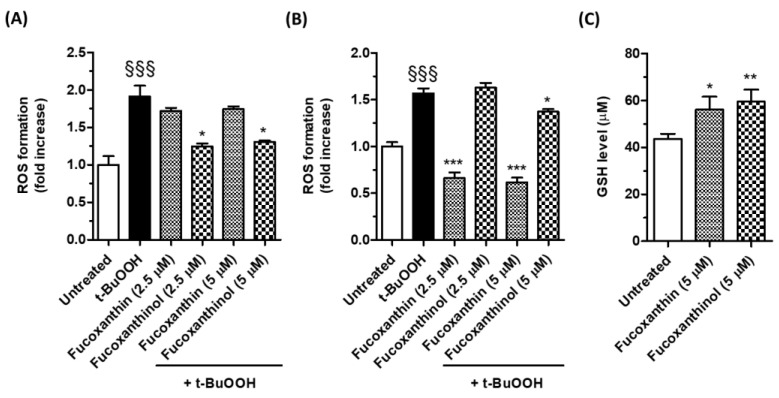
Effects of carotenoids on ROS formation in SH-SY5Y cells. (**A**) Cells were treated at the same time with the studied carotenoids (2.5–5 µM) and *t*-BuOOH (100 µM) for 30 min; (**B**) cells were treated for 24 h with the studied carotenoids (2.5–5 µM) and then with *t*-BuOOH (100 µM) for 30 min. After the different treatments, ROS formation was determined using the fluorescent probe DCFH-DA. Data are expressed as a fold increase in ROS formation induced by *t*-BuOOH and shown as mean ± SD of three independent experiments (§§§ *p* < 0.001 vs. untreated cells; * *p* < 0.05 and *** *p* < 0.001 vs. cells treated with *t*-BuOOH, one-way ANOVA with Tukey post hoc test); (**C**) cells were treated for 24 h with the studied carotenoids at a concentration of 5 µM. At the end of treatment, the GSH level was determined using the fluorescent probe MCB. Data are expressed as micromolar of GSH and shown as mean ± SD of three independent experiments (* *p* < 0.05 and ** *p* < 0.01 vs. untreated cells, one-way ANOVA with Tukey post hoc test).

To characterize the antioxidant profile of fucoxanthin and fucoxanthinol in SH-SY5Y cells, we evaluated the ability of these carotenoids to counteract the oxidative stress generated by *t*-BuOOH, a lipophilic organic peroxide [32]. Cells were treated with *t*-BuOOH (100 µM) at the same time or after 24 h of treatment with the carotenoids (2.5 and 5 µM). The ROS formation was determined using the fluorescent probe DCFH-DA. The treatment at the same time with all the concentrations of fucoxanthinol but not fucoxanthin significantly reduced the ROS formation induced by *t*-BuOOH in SH-SY5Y cells (Figure 3A). The treatment of the SH-SY5Y cells for 24 h with fucoxanthin before the treatment with *t*-BuOOH recorded a significant reduction at all concentrations (Figure 3B). The treatment with fucoxanthinol showed moderate inhibitory activity on ROS formation only at 5 µM (Figure 3B). These results reinforce the findings on TAA of the carotenoids in cell-free assay and in SH-SY5Y cells. The major antioxidant activity of fucoxanthinol in SH-SY5Y cells after the treatment at the same time with *t*-BuOOH could be the outcome of its high TAA at the extracellular level, whereas the better cellular uptake of fucoxanthin could explain its significant antioxidant activity after 24 h of treatment. These findings prompt us to evaluate the ability of the carotenoids also to increase the endogenous antioxidant defense. The SH-SY5Y cells were, therefore, treated for 24 h with fucoxanthin and fucoxanthinol at the concentration of 5 µM, and then the intracellular GSH levels were determined using the fluorescent probe MCB. GSH is the most abundant intracellular non-protein thiol and plays a crucial role in the maintenance of redox homeostasis in neurons [33]. Both the treatments with the studied carotenoids were shown to significantly increase the GSH levels in SH-SY5Y cells (Figure 3C). It is intriguing to note that although both the carotenoids resulted in similar increases in GSH, fucoxanthin exhibited a higher antioxidant activity than fucoxanthinol under the same treatment conditions. This phenomenon can be explained by the predominant free radical scavenging activity of fucoxanthin, which accumulates and distributes in cellular compartments. These results indicate that both fucoxanthin and fucoxanthinol can counteract the oxidative stress elicited by *t*-BuOOH in SH-SY5Y cells through their different antioxidant actions at the extracellular and cellular levels, respectively. Since *t*-BuOOH also has the ability to generate lipid peroxidation in brain tissues, these results are consistent with several in vivo models of cognitive impairment, subarachnoid hemorrhage, traumatic brain, and cerebral ischemia, demonstrating that fucoxanthin significantly decreases several parameters of oxidative damage, including the generation of malondialdehyde, a highly reactive product of lipid peroxidation that reflects the peroxidation of the biological membranes [3,4,34]. Currently, no studies have demonstrated the antioxidant activity of fucoxanthinol in in vitro and in vivo neuronal models. A recent study showed that fucoxanthinol reduced the lipid peroxidation induced by lipopolysaccharide (LPS) in murine RAW 264.7 macrophage cells at concentrations similar to those used in the experiments that support our findings [35]. Furthermore, fucoxanthinol has been found to decrease ROS formation in BV2 microglia cells stimulated by LPS [22].

### 3.2. Effects of Carotenoids on Neuronal Antioxidant Response

**Figure 4:**
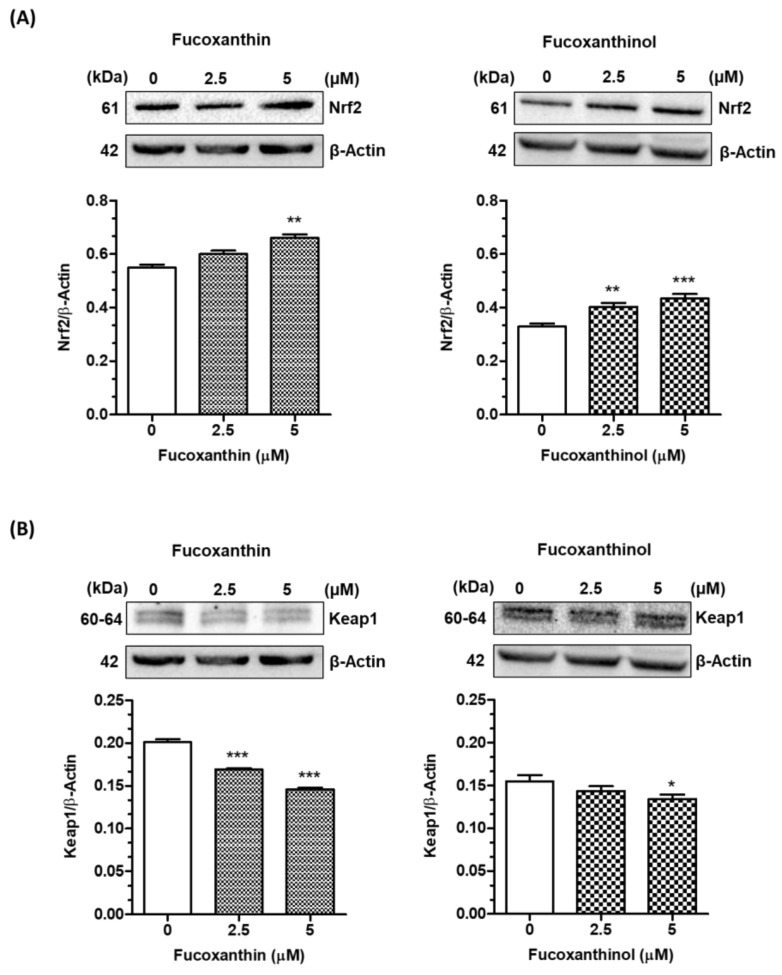
Carotenoids activate the Keap1/Nrf2/ARE pathway in SH-SY5Y cells. Cells were treated for 3 h with the studied carotenoids (2.5–5 µM). At the end of treatment, Nrf2 (**A**) and Keap1 (**B**) protein expression were determined using western blotting. Data are expressed as Nrf2/β-Actin or Keap1/β-Actin ratio and shown as mean ± SD of three independent experiments (* *p* < 0.05, ** *p* < 0.01 and *** *p* < 0.001 vs. untreated cells, one-way ANOVA with Tukey post hoc test).

The carotenoids fucoxanthin and fucoxanthinol improved the redox status of SH-SY5Y cells by reducing ROS formation and increasing GSH levels, suggesting the activation of neuronal antioxidant responses. Among antioxidant response pathways, we investigated the involvement of the Nrf2/ARE pathway, which plays a central defensive role in regulating the antioxidant response against OS. Under normal conditions, Nrf2 is bound to Keap1 in the cytoplasm. Nrf2 translocates to the nucleus and binds to the AREs located in the promoter regions of its target genes when the Nrf2/Keap1 complex is disrupted [25]. The ARE-controlled genes are responsible for synthesizing proteins that are crucial in maintaining the cellular redox status and protecting the cell against oxidative damage. These proteins include enzymes involved in the biosynthesis of GSH and Phase II detoxifying enzymes such as glutathione S-transferases (GST) and NAD(P)H: quinone oxidoreductase (NQO1) [36]. The treatment of SH-SY5Y cells for 3 h with fucoxanthin and fucoxanthinol significantly increased the total Nrf2 protein level at concentrations of 5 µM and 2.5 and 5 µM, respectively (Figure 4A). The same treatment with fucoxanthin recorded a significant reduction in Keap1 protein expression at all the tested concentrations, while the same treatment with fucoxanthinol reduced Keap1 protein expression only at the concentration of 5 µM (Figure 4B).

**Figure 5:**
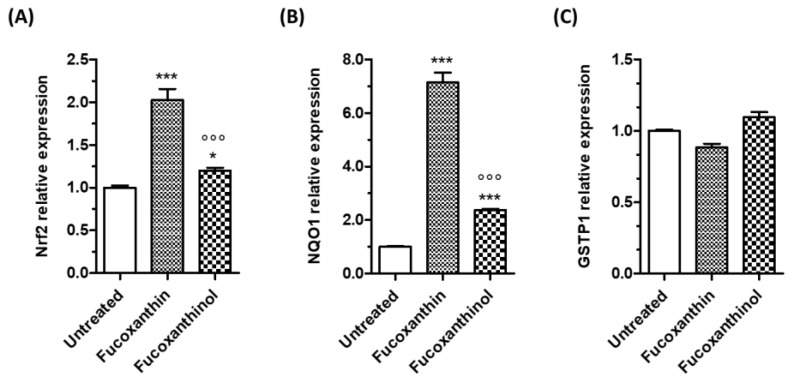
Effects of carotenoids on the mRNA expression of Nrf2 and Nrf2-target genes in SH-SY5Y cells. Cells were treated for 24 h with the studied carotenoids (5 µM). At the end of treatment, Nrf2 (**A**), NQO1 (**B**), and GSTP1 (**C**) gene expressions were determined using RT-PCR. Data are expressed as relative mRNA expression and shown as mean ± SD of three independent experiments (°°° *p* < 0.001 vs. cells treated with fucoxanthin; * *p* < 0.05 and *** *p* < 0.001 vs. untreated cells, one-way ANOVA with Tukey post hoc test).

To further confirm the increase in Nrf2 transcriptional activity upon treatment with carotenoids, the mRNA levels of Nrf2 and Nrf2-target genes, NQO1 and GSTP1, were then evaluated in SH-SY5Y cells (Figure 5). The treatment of SH-SY5Y cells for 24 h with both fucoxanthin and fucoxanthinol significantly increased Nrf2 and NQO1 gene expression at the concentration of 5 µM (Figure 5A,B). In particular, fucoxanthin showed a higher effect compared with that of fucoxanthinol. At the same experimental conditions, both the carotenoids had no effects on GSTP1 gene expression. It is known that GST is the main enzyme responsible for the removal of electrophilic products of lipid peroxidation formed in cellular membranes during oxidative stress by conjugating it with glutathione [37]. Therefore, in our experimental neuronal model, the carotenoids increase the GSH but do not complete their antioxidant action at membrane peroxidation level, confirming their main free radical scavenging activity as highlighted above. According to our findings on Nrf2 activation in SH-SY5Y cells through the use of carotenoids, a recent study has revealed that fucoxanthin can hamper the interaction between Keap1 and Nrf2 by forming hydrogen bonds with two crucial residues present in the Keap1 protein pocket—Arg415 and Tyr525 [38]. The inhibitory effects of fucoxanthin on Keap1-Nrf2 interaction resulted in activated Nrf2/Keap1/ARE pathway and increased the accumulation of nuclear Nrf2 protein, with a subsequent enhancement in the expression of Nrf2 target genes. Another study showed that fucoxanthin has a high affinity with Keap1, and it promotes the dissociation of the Keap1–Nrf2 complex, enhancing Nrf2 nuclear translocation in rat pheochromocytoma PC12 cells [39]. According to some studies, fucoxanthinol has been shown to activate the Nrf2/ARE signaling pathway, similar to its precursor, fucoxanthin. This activation results in the translocation of Nrf2 into the nucleus, which increases the expression of NQO1 and HO-1 protein in mouse macrophage RAW264.7 cells and murine microglial BV2 cells [22,24].

### 3.3. Neuroprotective Effects of Carotenoids against the Neurotoxicity Induced by AβO and 6-OHDA

Several in vitro and in vivo studies suggest that fucoxanthin may have neuroprotective effects against neurodegenerative events related to AD and PD. However, it is unclear if fucoxanthinol can have the same potential. Therefore, we compared the neuroprotective effects of fucoxanthin and fucoxanthinol against the neurotoxicity induced by AβO and 6-OHDA in SH-SY5Y cells using the MTT assay. The cells were treated with the neurotoxins at the same time or after 24 h of treatment with the carotenoids, similar to the evaluation of antioxidant activity against *t*-BuOOH. In particular, the 24 h treatment allowed us to assess whether the increased neuronal antioxidant response through the activation of the Nrf2/ARE pathway and the increase of GSH recorded by carotenoids could also prevent neurotoxicity caused by both neurotoxins.

**Figure 6:**
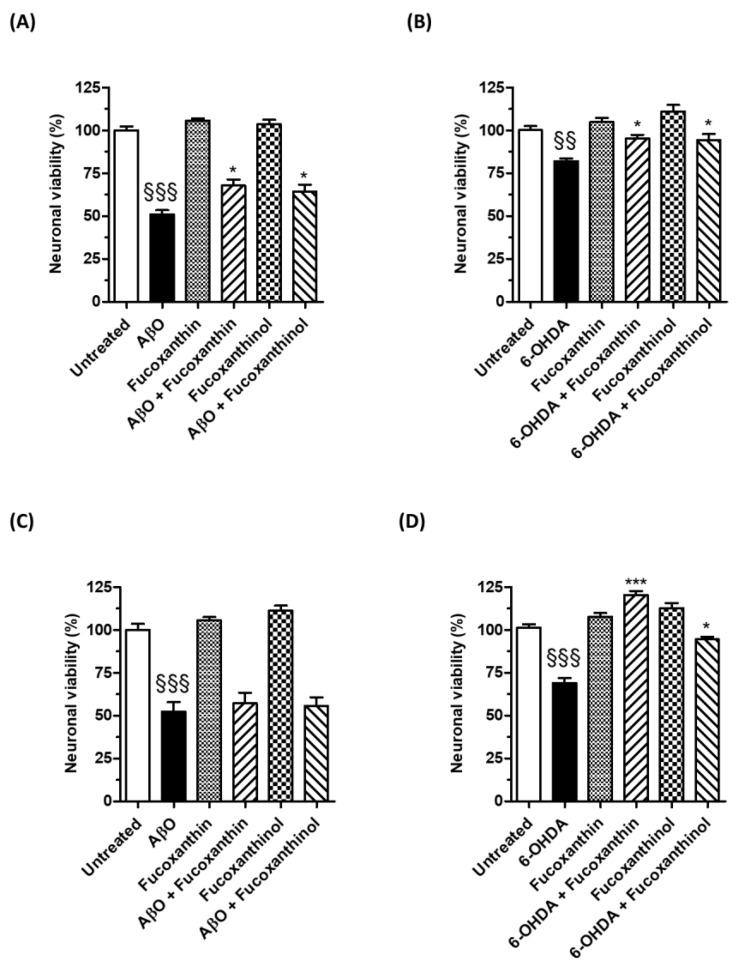
Neuroprotective effects of carotenoids against the neurotoxicity induced by AβO and 6-OHDA in SH-SY5Y cells. (**A**,**B**) Cells were treated at the same time with the studied carotenoids (5 µM) and AβO (10 µM) or 6-OHDA (100 µM) for 4 h and 2 h, respectively; (**C**,**D**) cells were treated with the studied carotenoids (5 µM) for 24 h and then with AβO (10 µM) or 6-OHDA (100 µM) for 4 h and 2 h, respectively. At the end of the treatments, the neuronal viability was determined using MTT assay. Data are expressed as percentages and shown as mean ± SD of three independent experiments (§§§ *p* < 0.001 and §§ *p <* 0.01 vs. untreated cells; * *p* < 0.05 and *** *p* < 0.001 vs. cells treated with AβO or 6-OHDA, one-way ANOVA with Tukey post hoc test).

As shown in Figure 6A,B, when SH-SY5Y cells were treated with the studied carotenoids (5 µM) and AβO (10 µM) or 6-OHDA (100 µM) for 4 and 2 h, respectively, the neurotoxicity elicited by neurotoxins was significantly reduced. The treatment of SH-SY5Y cells for 24 h with both carotenoids at the same concentration level was also shown to prevent the neurotoxicity mediated by 2 h of treatment with 6-OHDA (100 µM) (Figure 6D). Conversely, the treatment for 24 h with carotenoids did not modify the neurotoxic effects elicited by a 4 h treatment with AβO (10 µM) (Figure 6C), suggesting that the activation of the neuronal antioxidant response through the Nrf2/ARE pathway was not able to prevent AβO damage. These results are in accordance with the different neurotoxic mechanisms by which 6-OHDA and AβO lead to neuronal dysfunction and death. 6-OHDA forms an overload of free radicals and oxidative stress, while AβO causes a “permeabilization” of the cell membrane, resulting in irreversible loss of function [40,41].

Taken together, these results highlight the ability of both the carotenoids fucoxanthin and fucoxanthinol to directly counteract the neurotoxicity of AβO and strengthen the hypothesis that chemical interactions between these carotenoids and AβO block the access of AβO within the neuronal plasma membrane leading to neurotoxicity. In this regard, a recent study suggested a hydrophobic interaction between fucoxanthin and Aβ peptide, which might prevent the conformational transition and self-assembly of neurotoxic Aβ oligomers [5]. Another study showed that the inhibition of Aβ peptide by carotenoids may also be caused by the interaction between the hydroxyl groups of carotenoids and the acceptor groups of Aβ peptide [42]. Therefore, it is plausible that fucoxanthinol acts similarly to counteract the neurotoxicity mediated by AβO. Our results further highlight that fucoxanthinol shares the same neuroprotective effects as fucoxanthin against neurotoxicity generated by 6-OHDA. A previous study showed the ability of fucoxanthin to halt oxidative stress, the disruption of mitochondrial membrane potential, and apoptosis induced by 6-OHDA through the Nrf2/ARE pathway in PC12 cells [38]. Fucoxanthin also showed similar neuroprotective effects in zebrafish treated with 6-OHDA [38]. In this regard, no studies evaluated the neuroprotective effects of fucoxanthinol in experimental models of PD. Considering that the mitochondria are a critical intracellular target for ROS and electrophilic quinones generated by dopamine or through its 6-OHDA metabolite, the bioavailability of the carotenoids at neuronal levels plays an important role in their scavenger action directed against the ROS generated by 6-OHDA. In this context, our results show the ability of both the carotenoids fucoxanthin and fucoxanthinol to reach the neuronal cytoplasm where they can directly react with ROS, including hydrogen peroxide, superoxide anion, and hydroxyl radical, as well as dopamine and 6-OHDA-derived quinones. This prevents their attack on mitochondria and, ultimately, neuronal death [43]. Interestingly, the higher TAA found in the neuronal cytoplasm with fucoxanthin compared with fucoxanthinol could explain the difference in neuroprotective activity between the two carotenoids. These results show that both carotenoids can have cytoprotective effects at neuronal levels, but it is unclear if they reach the brain to exert neuroprotective effects. In this regard, several in vivo studies suggest that fucoxanthin may provide neuroprotective effects in the brain. A bioavailability study demonstrated that fucoxanthin could cross the blood–brain barrier and accumulate in the brains of mice at a dosage of 200 mg/kg [5]. Other studies have demonstrated that fucoxanthin can prevent stroke onset and cognitive impairment in rats and mice [10,44].

## 4. Conclusions

In this study, we compared the antioxidant activity of fucoxanthin and its metabolite fucoxanthinol in terms of free radical scavenging activity and induction of endogenous antioxidant response at the neuronal level and the neuroprotective effects against the neurotoxicity mediated by AβO and 6-OHDA in neuronal SH-SY5Y cells. The results showed the ability of both the carotenoids fucoxanthin and fucoxanthinol to cross the cell membrane and reach the cytoplasm, explaining the free radical scavenging activity at different subcellular levels. Notably, fucoxanthinol had higher and lower antioxidant activity than fucoxanthin at extracellular and cellular levels. Although both carotenoids exerted the ability to increase intracellular GSH through the Nrf2/Keap1/ARE pathway, our results suggest that the antioxidant activity of the carotenoids could be mainly attributed to their radical scavenging activity in neuronal membrane and cytoplasm where they accumulate. Interestingly, fucoxanthinol shared similar neuroprotective effects to fucoxanthin against neurotoxicity generated by AβO and 6-OHDA, suggesting a potential neuroprotective contribution to the action of fucoxanthin administered as a food supplement or nutraceutical in in vivo experimental model and humans. Many studies have shown that fucoxanthin is mainly converted into fucoxanthinol and amarouciaxanthin A in both animals and humans. Therefore, it is plausible that these metabolites accumulate in various tissues, contributing to the antioxidant and neuroprotective effects of fucoxanthin. While some studies have demonstrated that the oral administration of fucoxanthin can reverse acute neurodegenerative events in rats, further in vivo studies are needed to evaluate the bioavailability of fucoxanthin and its metabolites, including fucoxanthinol, at the brain level.

These findings can also be used to develop and conduct human intervention studies. These studies should focus on studying the bioavailability of fucoxanthin from food supplements and providing further evidence of its potential health benefits for the brain. Although the research on humans is still in its early stages, several studies are ongoing and have recorded the potential contributions of carotenoids to the prevention and treatment of eye diseases and the reduction of several risk factors for diabetes and cardiovascular diseases, including hyperglycemia, hypercholesterolemia, and oxidative stress [45]. Among the studied carotenoids, xanthophylls such as lutein and zeaxanthin show the potential to prevent cognitive decline, suggesting their beneficial effects on brain health. Interestingly, these xanthophylls share common physical and chemical characteristics, as well as antioxidant properties of fucoxanthin, prompting the definition of nutritional strategies that use fucoxanthin as a functional food ingredient to prevent oxidative stress, which is implicated in neurological disorders and neurodegenerative diseases [46].

## Data Availability

The data presented in this study are available on request from the corresponding author.

## Supplementary Materials

The following supporting information can be downloaded at: https://www.mdpi.com/article/10.3390/cimb46060357/s1, Figure S1: Neurotoxicity of the carotenoids in SH-SY5Y cells. Cells were treated with various concentrations (2.5-40 μM) of the studied carotenoids for 24 h. At the end of treatments, the neuronal viability was determined by MTT assay. Data are expressed as percentages and shown as mean ± SD of three independent experiments (*** *p* < 0.001 vs. untreated cells, one-way ANOVA with Tukey post hoc test).
