# Supplementary material for: Antioxidant and Neuroprotective Effects of Fucoxanthin and Its Metabolite Fucoxanthinol: A Comparative In Vitro Study
Source: Curr Issues Mol Biol. 2024 Jun 14;46(6):5984–98. doi: 10.3390/cimb46060357 (PMC11202671; doi:10.3390/cimb46060357)
Supplement: Supplementary file 1 [file cimb-46-00357-s001.zip › cimb-2985977-supplementary.pdf]

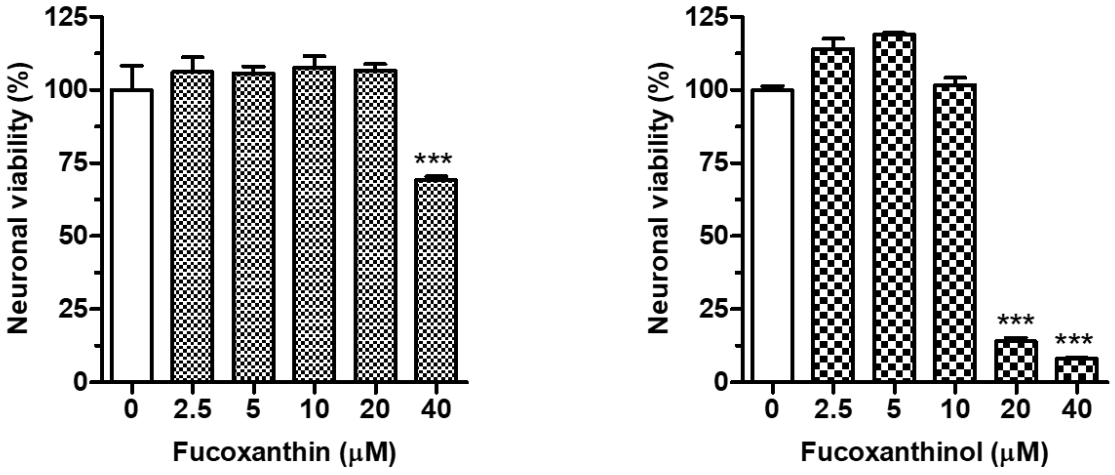

Figure S1. Neurotoxicity of the carotenoids in SH-SY5Y cells. Cells were treated with various concentrations (2.5-40 μM) of the studied carotenoids for 24 h. At the end of treatments, the neuronal viability was determined by MTT assay. Data are expressed as percentages and shown as mean ± SD of three independent experiments (*** $p < 0.001$ vs. untreated cells).
